# Supplementary material for: Limited predictive value of the gut microbiome and metabolome for response to biological therapy in inflammatory bowel disease
Source: Gut Microbes. 2024 Aug 21;16(1):2391505. doi: 10.1080/19490976.2024.2391505 (PMC11340771; doi:10.1080/19490976.2024.2391505)
Supplement: Supplemental Material [file KGMI_A_2391505_SM9408.zip › Supplementary figure S1.docx]

**Supplementary Figure S1**

**
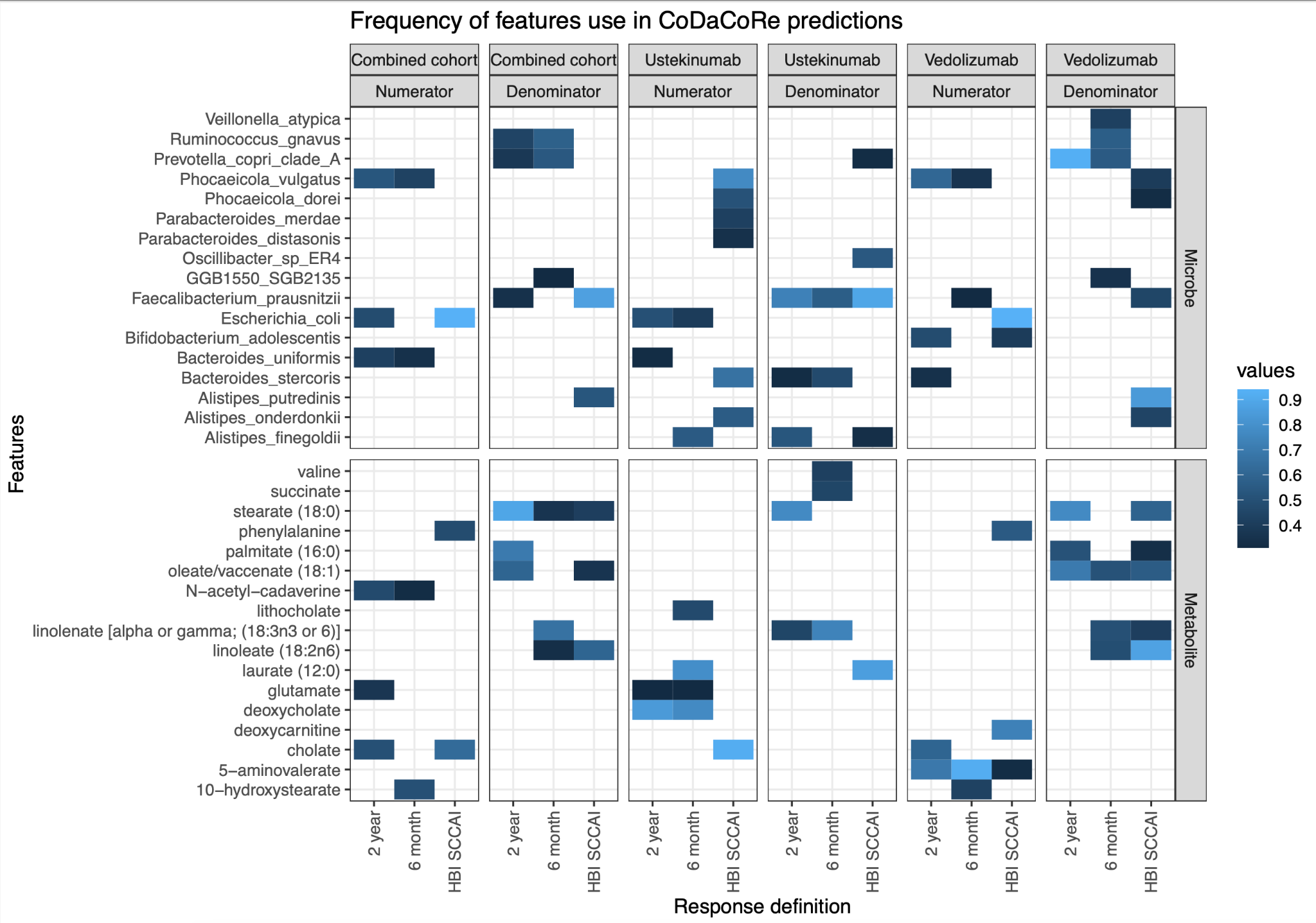
**

**Frequency of features used in the CoDaCoRe predictions over all medication and response definitions**

Shown are the predictors from the CoDaCoRe permutation analysis. Only features selected more than 10% of the time are shown. For each cohort the Numerator and Denominator are shown and for each response definition. Features highlighted in the numerator are more abundant in responders and features in the denominator are more abundant in non-responders.
